# Organogenesis *versus* somatic embryogenesis pathway efficiencies in *in vitro* propagation of white and water yams

**DOI:** 10.1007/s11627-023-10397-7

**Published:** 2023-11-27

**Authors:** Chukwunalu O. Ossai, Morufat O. Balogun, Norbert G. Maroya

**Affiliations:** 1 Department of Crop Protection and Environmental Biology, University of Ibadan, Ibadan, Nigeria; 2 International Institute of Tropical Agriculture, IITA, Ibadan, Nigeria

**Keywords:** Yam seed system, Yam species, Organogenesis, Somatic embryogenesis, Yam Plantlet

## Abstract

The primary goal of this study was to compare the multiplication rates of yam varieties propagated through organogenesis and somatic embryogenesis (SE). Callus was induced from axillary bud explants of three genotypes of *Dioscorea rotundata* (Asiedu, Ekiti2a, and Kpamyo) and two genotypes of *Dioscorea alata* (Swaswa and TDa2014) cultured in Murashige and Skoog (MS) medium containing 9.1 µM 2,4-dichlorophenoxylacetic acid and 5.4 µM naphthaleneacetic acid. Plantlets were regenerated in MS containing 4.4 µM benzylaminopurine and 34 µM uniconazole-P through SE. Single-node cuttings of the five genotypes were grown in MS for 8 wk *via* organogenesis. The SE and organogenesis regenerants were acclimatized and potted in a 2 (propagation techniques (PTs)) × 5 (genotypes) factorial arranged in a completely randomized design (*r* = 10). The multiplication ratios (MR), number of tubers (NoT) of the SE, and organogenesis regenerants were collected and analyzed using ANOVA, and means were separated using DMRT (*P* ≤ 0.05). The SE and organogenesis MR ranged from 1:2 (TDa2014) to 1:8 (Asiedu) and 1:4 (Asiedu) to 1:5 (Ekiti2a and TDa2014), respectively. The NoT differed among genotypes, ranging from 1.15 ± 0.49 (Swaswa) to 2.45 ± 1.39 (Asiedu), and between PTs, ranging from 1.42 ± 0.70 (SE) to 1.86 ± 1.11 (organogenesis). The optimum propagation pathway was genotype-specific.

## Introduction

Yam (*Dioscorea* sp.) is a multi-species tuber crop cultivated in different parts of the world (Adeigbe *et al.* 2015). Over 93% of yam production worldwide occurs in the yam belt region of West Africa with Nigeria alone producing about 70% of the world’s total (FAO 2018). In 2012, yam produced in Nigeria was valued at $7.75 billion, and it was cultivated from 2.9 million hectares of land. This contributed over 12% of total gross domestic products in Nigeria with an estimated profit of over US $13,000 per hectare harvested (IITA 2013). However, despite Nigeria being the leading producer of yam, the country is not among the top ten exporters of yam, and its availability is still below the consumer demand, implying that bulk of the yam produced in Nigeria were consumed within the country. This is due to predominantly informal (farmer-saved seeds) seed system characterized by low propagation ratio, scarcity of clean seed yam, and uncontrolled sprouting after dormancy break, which causes postharvest losses (Balogun *et al.* 2018).

Different propagation methods have been adopted over the years to address the problem of low propagation ratio of yam, each with its pros and cons. In the traditional system of yam cultivation, tubers weighing up to 200 g are used for planting, and in some cases, tuber sizes above 200 g are cut into smaller sizes (minisetting) for planting (Balogun 2009; Otoo *et al.* 2016). In a well-developed formal seed system, minitubers should be specifically reserved for planting while bigger tubers (ware yam) serve for consumption (Aighewi *et al.* 2021). Also in the traditional system, a milking method of harvest is done (Okoli *et al.* 1982), which allows the harvesting of whole tuber at 66% growing period and seed yam at full senescence, thus doubling the propagation ratio to 1:2.

Other propagation methods developed include the partial sectioning technique (Nwosu 1975), the minisett technique (Okoli *et al.* 1982; Aighewi *et al.* 2014), and the vine rooting technique (Acha *et al.* 2004). Despite the progress reported in the above propagation methods over the years, the propagation ratio of yam cannot be compared to the 1:300 obtained in cereals (Mbanaso *et al.* 2011). Tissue culture is one such method with rapid means of propagating yam *in vitro*, having the ability to clean yam from pathogens. There are several reports of its use in the propagation of different yam species using different plant parts (Shu *et al.* 2005). The most common method in yam propagation *in vitro* has been through the regeneration of plantlets from organs with pre-existing meristems (organogenesis) (Balogun and Gueye 2013).

Recent reports on protocols for inducing somatic embryos from organs without pre-existing meristems and subsequently regenerating plantlets from them, a system known as somatic embryogenesis (SE), have offered a new look at the yam propagation ratio (Dodeman *et al.* 1997; Ossai *et al.* 2018). This system is regarded as the peak expression of cell totipotency (Gutiérrez-Mora *et al.* 2012) in plants, and SE has been applied in over 100 plant species (Merkle 1997). Unlike organogenesis, the success in producing transgenic plants is limited to SE that has an intervening callus phase (Fehér 2008). However, both SE and organogenesis can be induced in same tissue culture conditions (Castillo *et al.* 2000) which makes differentiating SE from organogenesis complicated and in most cases requires histological analysis of the processes that suggest embryo-like origin in SE (Gaj 2004). In yam, reports on the applicability of SE have been reported in *Dioscorea floribunda* (mule’s hoof), *Dioscorea composite* (barbasco), *Dioscorea alata* (water yam), *Dioscorea bulbifera* (aerial yam), *Dioscorea rotundata* (white yam), and *D. zingiberensis* (Chinese yam) (Shu *et al.* 2005). Despite the success reported, the SE system is limited to improved varieties and genotype-specific (Suarez *et al.* 2011; Manoharan *et al.* 2016; Balogun* et al.* 2017). Hence, there is a need to evaluate it in more genotypes including landraces for wide applicability (Landi and Mezzetti 2005). In addition, there is no report on the post-flask performance of yams regenerated through SE in comparison with the more optimized *in vitro* propagation method (organogenesis). This study, therefore, reported an evaluation of multiplication rates of improved white and water yam genotypes a Nigerian white yam landrace to organogenesis and SE, and the post-flask performances of the regenerants towards effective out-scaling of the production of yam plantlets *in vitro*.

## Materials and Methods

### Study location/source of planting material

The experiment was carried out at the Tissue Culture Laboratory, Cell Biology Unit of the Bioscience Center of IITA under the Yam Improvement for Income and Food Security in West Africa Phase II (YIIFSWA-II) project. Virus-free plantlets of three genotypes of *Dioscorea rotundata* Poir. (Asiedu, Ekiti2a, and Kpamyo) and two genotypes of *Dioscorea alata* L. (Swaswa and TDa2014) were maintained *in vitro* in yam multiplication medium (YMM). The YMM contained Murashige and Skoog (MS; Murashige 1974), 100.0 mg L^−1^ myo-inositol, 30.0 g L^−1^ sugar, 1.0 mg L^−1^ kinetin, 20.0 mg L^−1^ l-cysteine, 7.0 g L^−1^ agar, and 1.0 g L^−1^ activated charcoal (AC) at a pH of 5.7 ± 0.01 (Balogun *et al.* 2017) which were sourced from the YIIFSWA Project of IITA. All the reagents and plant growth regulators (PGRs) used in this study were sourced from Bristol Scientific, a Sigma-Aldrich distributor in Lagos, Nigeria.

### Plantlet propagation through organogenesis

Five single-node cuttings of above *Dioscorea rotundata* (Asiedu, Ekiti2a, and Kpamyo) and *Dioscorea alata* (Swaswa and TDa2014) genotypes were subcultured into plastic vented vessels containing 70 mL of YMM. The cultures were arranged in a completely randomized design (CRD) and replicated three times in a chamber conditioned at 25 ± 1°C and 16-h photoperiod (4000 lx) for eight (8) wk. At the 8th wk of culture, the number of nodes per plantlet was recorded.

### Plantlet propagation through somatic embryogenesis-callus induction

Axillary bud explants (0.1 to 0.5 cm), excised from 2-wk-old plantlets (young plant) of Kpamyo, Asiedu, Ekiti2a, Swaswa, and TDa2014 (4 explants per Petri plate), were cultured into YMM medium supplemented with 9.1 µM 2,4-dichlorophenoxylacetic acid (2,4-D) and 5.4 µM naphthaleneacetic acid (NAA) under laminar flow hood. The cultures were incubated in a dark condition for 4 wk for callus induction.

### Somatic embryo formation and maturation

The induced calluses were transferred to a PGR-free YMM for embryo formation. The cultures were incubated at 16-h photoperiod (4000 lx) and 25 ± 1°C for 3 wk. At 2 wk of callus transfer to PGR-free YMM, the embryogenic development stage (either globular, heart shaped, torpedo, and cotyledonal) was confirmed by viewing the calluses with a stereo photomicroscope (Koolertron) equipped with a digital 5-inch LCD 1080p at 40 × at the Virology Laboratory Unit of IITA, Ibadan, Nigeria.

### Plantlet regeneration

The somatic embryos (at different developmental stages) at 3 wk of callus transfer to PGR-free YMM were thereafter transferred into Petri plates (16.7 mL per plate) containing plantlet regeneration medium (PRM) consisting of YMM supplemented with 4.4 µM of BAP and 34 µM uniconazole-P. The medium was autoclaved at 121°C and 15 Psi for 15 min. The cultures were kept at 16-h photoperiod (4000 lx) at 25 ± 1°C.

### Histological studies of somatic embryogenesis stages

This was carried out at the Department of Botany, University of Ibadan, Nigeria. Callus tissues were sampled at 4, 6, and 8 wk of culturing (WOC) and fixed in formaldehyde:acetic acid:alcohol mixed by volume at a ratio of 5:5:90 mL and kept at 10°C for an interval of 48 h before dehydration in 70% ethanol. After dehydration, they were embedded in pawpaw tissues (sourced from the Anatomy Laboratory, Department of Botany, University of Ibadan, Ibadan, Oyo State, Nigeria) and sectioned longitudinally using a rotary microtome (Leica RM 2155-UK), stained with 0.05% toluidine blue (sourced from Bristol Scientific, Lagos, Nigeria) for 4 min, and mounted on cytological glass slides (Manoharan *et al.* 2016). Images were obtained with the aid of an Olympus light microscope (40 ×).

### Post-flask evaluation of plantlets produced through somatic embryogenesis and organogenesis

The *in vitro* produced plantlets were acclimatized following the protocol developed by Balogun *et al.* (2017). Ten (10) plantlets each of Asiedu, Ekiti2a, Kpamyo, Swaswa, and TDa2014 produced through somatic embryogenesis and organogenesis, respectively, were hardened. The roots of the plantlets were rinsed in sterile distilled water before planting them in a rectangular bowl containing moistened coco peat (500 g). The top of the bowl was covered with a laser perforated nylon sheet (http://www.vivi.nu) that controls aeration. The plants were kept in the screen house at 28 ± 1°C with an average daily light intensity of 3916.6, 7410, and 980 mmol m^−2^ s^−1^ at 10:00 am, 2:00 pm, and 6:00 pm, respectively. The laser perforated nylon sheet was uncovered after 2 wk of acclimatization, and the acclimatized plantlets were transferred to plastic pots filled with 4 kg sterilized fertigated topsoil with perforations on the base for water drain-off to prevent water logging. A pot contained one plant per genotype. The plants were watered every 4 d while they were fertigated with nutrient solution (0.04 g L^−1^ ammonium nitrate, 0.23 g L^−1^ potassium nitrate, 0.24 g L^−1^ calcium nitrate, 0.06 g L^−1^ magnesium sulfate, 0.28 g L^−1^ potassium sulfate, 0.20 g L^−1^ potassium phosphate, 0.06 g L^−1^ triple super phosphate, and 0.01 g L^−1^ Terratiga chelate) (Maroya *et al.* 2017) every 4 d until harvest (7 mo after planting).

### Experimental design, data collection, and statistical analysis

For the SE regeneration, the experiment was arranged in a completely randomized design (CRD) and replicated 3 times. Each replicate comprised of three Petri plates, each with 5 explants per Petri plate per genotype. The following data were collected per explant per genotype: (1) percentage of explant forming callus, (2) number of days to callus formation, (3) number of days from explant incubation to plantlet regeneration per explant, (4) number of plantlets regenerated per explant, and (5) number of roots formed. Data collected were analyzed using ANOVA (SAS 9.0 version), and the genotypic means were separated using the Duncan multiple range test (DMRT) at *P* ≤ 0.05. For the calculation of propagation rate of both organogenesis and somatic embryogenesis regeneration pathways, the minimum number of nodes, maximum number of nodes, average number of nodes, and days from culture to subculture were collected to compare the multiplication efficiency within a 16-wk period. For the post-flask study, the experiment was a 5 (genotypes) × 2 (propagation techniques: organogenesis and SE) factorial in a CRD replicated 10 times. Data were collected on the number of new leaves formed after hardening, number of nodes after hardening, plant height (cm) after hardening, number of nodes at 2, 4, 6, and 8 wk after potting, number of tubers at harvest, and tuber weights (g) per plant at harvest (7 mo after potting). The data were analyzed using ANOVA, and differences in treatment means were separated using the DMRT at *P* ≤ 0.05.

## Results

### Response of selected genotypes of white and water yam to somatic embryogenesis

The shortest days to callus formation (15 d) were observed in Asiedu, which was significantly faster than the days taken by Swaswa by 5 d and TDa2014 by 11 d (Table 1). There were no differences between Kpamyo, Asiedu, and Ekiti2a in the percentage of callus formation with ≥ 95%. This is 47% higher than Swaswa and 62% higher than TDa2014. The number of plantlets regenerated was significantly higher than the rest of the genotypes. The plantlets were regenerated in Ekiti2a within a period of 58 d and this was significantly faster than the days from culture to regeneration in Asiedu and Swaswa by 8 and 10 d, respectively. The number of roots formed by Asiedu, Kpamyo, and Ekiti2a was not significantly different from each other, but they were statistically higher than Swaswa and TDa2014.

**Table 1.** Mean values of somatic embryogenesis traits in five genotypes of *Dioscorea rotundata* Poir. and *Dioscorea alata* L

Genotypes	NDTCF	% CF	NPR	NDTPR	NRF
Asiedu	14.63 ± 3.60c	96.67 ± 7.78a	4.83 ± 3.33b	66.48 ± 10.47a	3.33 ± 1.61a
Kpamyo	16.85 ± 5.63bc	100.00 ± 0.00a	4.08 ± 2.57b	63.29 ± 9.16ab	4.08 ± 1.73a
Ekiti2a	16.29 ± 4.14bc	95.00 ± 9.05a	7.92 ± 3.97a	58.28 ± 6.88b	3.83 ± 1.75a
Swaswa	20.75 ± 3.95b	47.92 ± 25.00b	1.67 ± 1.35c	68.89 ± 12.31a	1.50 ± 1.33b
TDa2014	26.08 ± 2.81a	33.33 ± 12.31b	1.58 ± 1.38c	65.78 ± 5.84ab	1.25 ± 0.92b

Means with the same *letter* down the group are not significantly different from each other. *NDTCF* number of days to callus formation, *% CF* percentage callus formation at 4 wk of culturing, *NPR* number of plantlets regenerated at 12 wk of culturing, *NDTPR* number of days to plantlet regeneration, *NRF* number of roots formed at 12 wk of culturing

### Relative multiplication rates of yam propagated *via* somatic embryogenesis and organogenesis

The average number of nodes obtained from the cultured explant at sixteen (16) wk of culturing *via* organogenesis ranged from 16 nodes (Asiedu) to 25 nodes (Ekiti2a and TDa2014); however, through SE, the average number of nodes ranged from 10 nodes (TDa2014) to 21 nodes (Asiedu) (Table 2). Between the organogenesis and SE propagation methods, the average number of nodes obtained in the five genotypes *via* organogenesis (21) was 24% higher than the average nodes obtained in the genotypes *via* SE (16).

**Table 2.** Propagation rates of five *Dioscorea rotundata* Poir. and *Dioscorea alata* L. genotypes regenerated through somatic embryogenesis and organogenesis

Genotypes	Organogenesis				Somatic embryogenesis			
	Min*N*	Max*N*	ANN	Age (wk)	Min*N*	Max*N*	ANN	Age (wk)
Asiedu	9	15	16	16	12	27	21	16
Ekiti2a	9	49	25	16	9	24	15	16
Kpamyo	16	25	21	16	3	24	15	16
Swaswa	9	25	18	16	9	30	19	16
TDa2014	9	36	25	16	6	15	10	16
Average	10.4	30	21	16	7.8	24	16	16

*MinN* minimum number of nodes, *MaxN* maximum number of nodes, *ANN* average number of nodes, *DTS* days from culture to subculture

### Histology of callus tissues at 4, 6, and 8 wk of culturing

**Figure 1.**
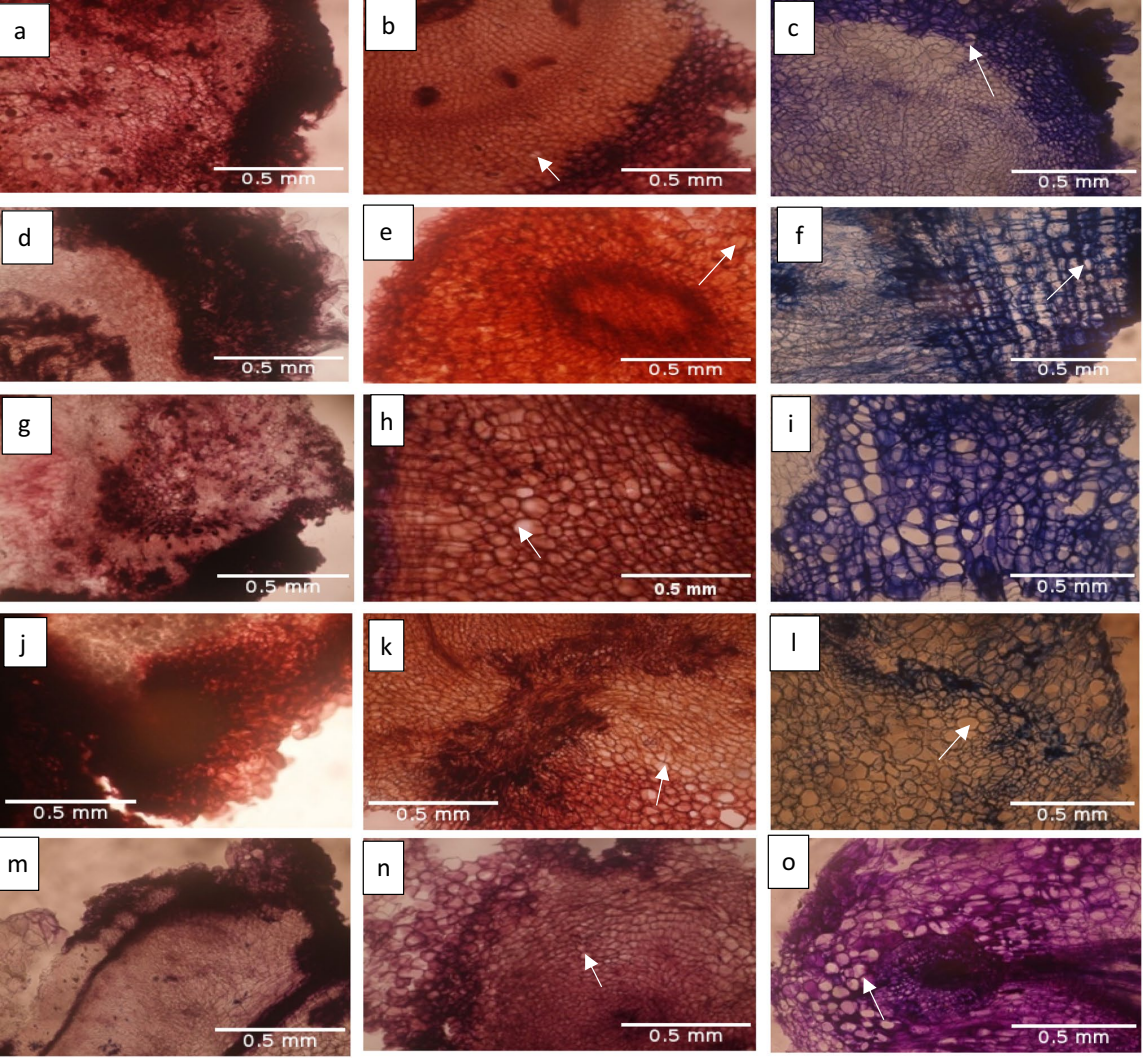
Photomicrographs of somatic embryogenesis phases of white and water *Dioscorea rotundata* Poir. and *Dioscorea alata* L. at × 10. Keys: (*a*–*c*) Callus sections of Swaswa at 4, 6, and 8 wk of culturing respectively. (*d–f*) Callus sections of TDa2014 at 4, 6, and 8 wk of culturing respectively. (*g–i*) Callus sections of Asiedu at 4, 6, and 8 wk of culturing respectively. (*j–l*) Callus sections of Kpamyo at 4, 6, and 8 wk of culturing respectively. (*m–o*) Callus sections of Ekiti2a at 4, 6, and 8 wk of culturing respectively. *Arrow*: Vascular tissue arrangement.

The photomicrograph of the three stages (4, 6, and 8 WAI) of somatic embryogenesis processes of the three white yam and two water yam genotypes revealed that at 4 WAI, there was no definite tissue arrangement in all the genotypes, rather an active cellular proliferation with no definite tissue arrangement pattern. However, at 6 and 8 WAI, there was structural tissue arrangement with visible vascular bundles (Fig. 1).

### Post-flask performance of somatic embryogenesis– and organogenesis-produced plantlets of white and water yam

Asiedu produced the highest number of new leaves (4.00 ± 2.34) during acclimatization, which was not significantly different from Ekiti2a (3.70 ± 2.15), Kpamyo (3.20 ± 1.94), and Swaswa (3.20 ± 1.23) but was significantly higher than TDa2014 (2.40 ± 1.47). The number of nodes produced by Asiedu (6.60 ± 3.69) was not significantly different from TDa2014 (6.55 ± 2.67), but they were both significantly higher than Kpamyo (4.45 ± 2.19). The height (10.02 ± 7.07) of Asiedu was significantly higher than the rest of the genotypes evaluated. The survival rates of the plantlets produced through somatic embryogenesis and organogenesis were not significantly different. However, the number of new leaves (3.96 ± 1.89), number of nodes (6.44 ± 2.67), and the height (8.73 ± 2.26 cm) of the plants produced through somatic embryogenesis were significantly higher than plantlets produced *via* organogenesis with 2.64 ± 1.71, 5.36 ± 2.83, and 5.33 ± 2.26 cm for number of new leaves, number of nodes, and the plant height, respectively. The interaction between genotypes and propagation pathways was significant for the number of leaves, number of nodes, and plant height except for the hardening survival rate (Table 3). The number of nodes produced after potting increased gradually from 4 to 8 wk in all the genotypes. At eight (8) WAP, Asiedu had the highest number of nodes (75.70 ± 35.16), which was significantly higher than the rest of the genotypes. The number of tubers (2.45 ± 1.39) produced by Asiedu and the average tuber weight (68.36 ± 20.79), respectively, were significantly higher than the rest of the genotypes. The number of tubers produced by TDa2014 (1.75 ± 0.72) was not significantly different from Kpamyo (1.55 ± 0.60) and Ekiti2a (1.30 ± 0.73) but was significantly (*P* ≤ 0.05) higher than Swaswa (1.15 ± 0.49), while the weight of tubers produced by Asiedu (68.36 ± 20.79) was significantly (*P* ≤ 0.05) higher than the other genotypes. However, the number of tubers produced by the plantlets raised through organogenesis (1.86 ± 1.11) was significantly higher than the somatic embryogenesis–raised plantlets (1.42 ± 0.70). The interaction between the genotypes and propagation pathways was significant (*P* ≤ 0.05) for the number of nodes and tuber weight (Table 4).

**Table 3.** *Ex* *vitro* performance of five (5) *Dioscorea rotundata* Poir. and *Dioscorea alata* L. genotypes propagated *via* somatic embryogenesis and organogenesis at 2 wk after acclimatization

	Survival rate (%)	Number of new leaves	Number of nodes	Plant height (cm)
Genotypes				
Asiedu	95.00 ± 0.09a	4.00 ± 2.34a	6.60 ± 3.69a	10.02 ± 7.07a
Kpamyo	90.00 ± 0.13a	3.20 ± 1.94ab	4.45 ± 2.19b	5.27 ± 2.49c
Ekiti2a	100.00 ± 0.00a	3.70 ± 2.15a	5.95 ± 2.63ab	5.63 ± 1.66bc
Swaswa	100.00 ± 0.00a	3.20 ± 1.23ab	5.96 ± 2.21ab	6.93 ± 3.40bc
TDa2014	100.00 ± 0.00a	2.40 ± 1.47b	6.55 ± 2.67a	7.33 ± 2.2.43b
Propagation pathway (PP)				
SE	100.00 ± 0.00a	3.96 ± 1.89a	6.44 ± 2.67a	8.73 ± 2.26a
Org	94.00 ± 0.10a	2.64 ± 1.71b	5.36 ± 2.83b	5.33 ± 2.26b
Genotypes by PP	ns	*	*	*

Means with the same *letter* down the group are not significantly different from each other at 5% level of significance. *SE* somatic embryogenesis, *Org* organogenesis, *Ns* not significant. *Significant at 5% significance level

**Table 4.** Relative post-flask growth performance and tuber yield of 5 *Dioscorea rotundata* Poir. and *Dioscorea alata* L. genotypes propagated *via* somatic embryogenesis and organogenesis at 4 to 8 wk after acclimatization and at harvest

	Number of nodes			Number of tubers	Tuber weight (g)
	4 wk	6 wk	8 wk		
Genotypes					
Asiedu	30.60 ± 14.17a	43.65 ± 21.78a	75.70 ± 35.16a	2.45 ± 1.39a	68.36 ± 20.79a
Kpamyo	19.50 ± 7.29b	27.80 ± 9.46b	48.25 ± 14.76b	1.55 ± 0.60bc	46.68 ± 14.05b
Ekiti2a	14.20 ± 9.78b	17.30 ± 10.54c	31.60 ± 21.30c	1.30 ± 0.73bc	33.72 ± 20.56c
Swaswa	16.30 ± 6.67b	28.00 ± 13.25b	38.90 ± 14.41bc	1.15 ± 0.49c	18.86 ± 8.55d
TDa2014	16.30 ± 6.78b	28.05 ± 13.29b	45.45 ± 13.14bc	1.75 ± 0.72b	27.44 ± 10.91 cd
Propagation pathway (PP)					
SE	22.26 ± 12.08a	31.72 ± 18.89a	51.06 ± 30.25a	1.42 ± 0.70b	36.38 ± 23.40a
Org	16.58 ± 8.82b	26.60 ± 13.07a	44.90 ± 20.31a	1.86 ± 1.11a	41.64 ± 22.99a
Genotypes by PP	*	*	*	*	*

Means with the same *letter* down the group are not significantly different from each other at 5% level of significance. *SE* somatic embryogenesis, *Org* organogenesis. *Significant at 5% significance level

### Variations in post-flask growth and yield performance among genotypes of white and water yam produced *via* different micropropagation pathways

**Figure 2.**
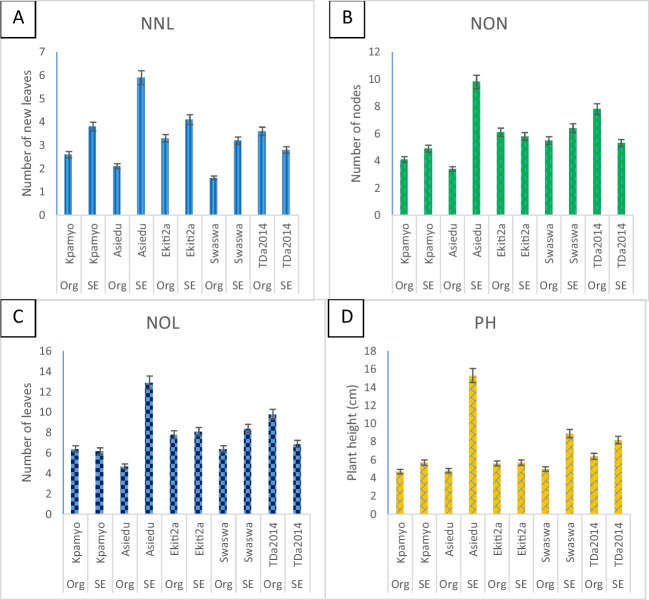
*(A*) Number of new leaves, (*B*) number of nodes, (*C*) number of leaves, and (*D*) plant height of white and water *Dioscorea rotundata* Poir. and *Dioscorea alata* L. plantlets produced through somatic embryogenesis and organogenesis after 2 wk of acclimatization**.** Keys: NNL number of new leaves, NON number of nodes, NOL number of leaves, PH plant height, Org organogenesis, SE somatic embryogenesis.

The average number of new leaves, number of nodes, number of leaves, and plant height after 2 wk of acclimatization varied across genotypes and source of plantlets (organogenesis and SE) (Fig. 2). In Kpamyo, the number of new leaves produced by the plantlets regenerated through SE (3.8) was 32% more than the new leaves produced by Kpamyo plantlets regenerated through organogenesis (2.6). In Asiedu, the number of new leaves produced by the plantlets regenerated through SE (5.9) was 64% more than the new leaves produced by Kpamyo plantlets regenerated through organogenesis (2.1). New leaf production in Ekiti2a multiplied *via* SE (4.1) was 20% more than organogenesis-raised plantlets (3.3). In Swaswa, the new number of leaf production in the SE-raised plantlets (3.2) was 50% more than the organogenesis-raised plantlets (1.6). However, in TDa2014, the number of new leaves produced by the organogenesis-raised plantlets (3.6) was 22% more than the SE-raised plantlets (2.8). On the NON, in Kpamyo, Asiedu, and Swaswa, the nodal production in the SE-raised plantlets was 16%, 64%, and 14%, respectively, more than the organogenesis-raised plantlets. However, in Ekiti2a and TDa2014, the NON produced in the organogenesis-raised plantlets were 5% and 32% higher than the SE-raised plantlets, respectively. In the NOL produced by Kpamyo and TDa2014, the organogenesis-raised plantlets were 3% and 30%, respectively, higher than the SE-raised plantlets. On the plant height, the SE-raised plantlets were taller than the organogenesis-raised plantlets in Kpamyo, Asiedu, Ekiti2a, Swaswa, and TDa2014 by 14%, 69%, 1%, 43%, and 22%, respectively.

**Figure 3.**
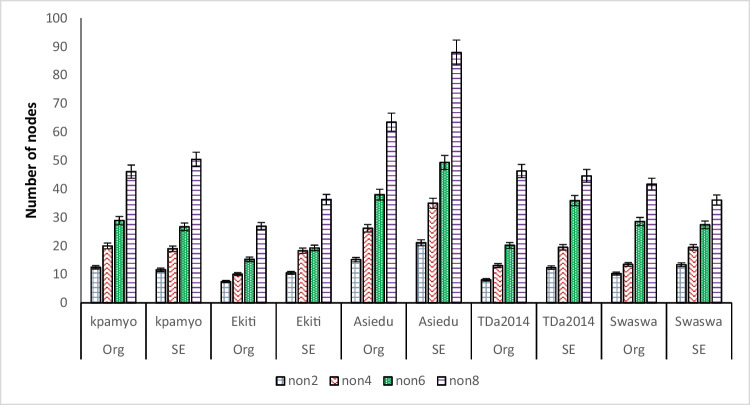
Average number of nodes produced by somatic embryogenesis– and organogenesis-raised plantlets of white and water *Dioscorea rotundata* Poir. and *Dioscorea alata* L. in post-flask. Keys: NON2–NON8 number of nodes at 2, 4, 6, and 8 wk after potting, Org organogenesis, SE somatic embryogenesis.

**Figure 4.**
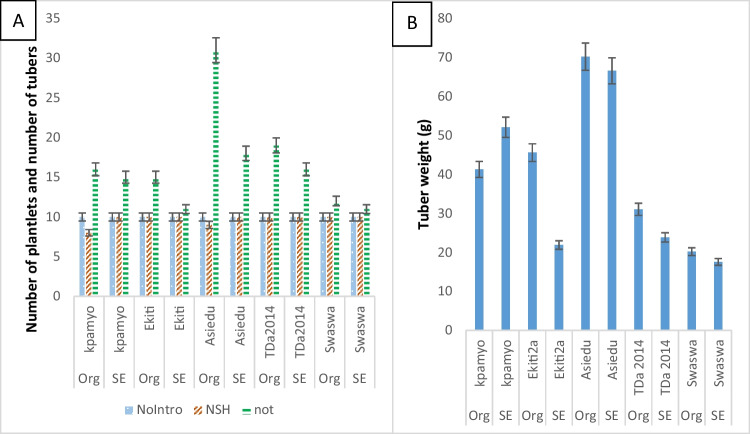
*(A*,* B)* Post-flask survival and tuber yield of *in vitro* produced plantlets of white and water *Dioscorea rotundata* Poir. and *Dioscorea alata* L. Keys: NoIntro number of plantlets introduced to hardening, NSH number of plantlets successfully hardened, NOT number of tubers harvested after 7 mo, Org organogenesis, SE somatic embryogenesis.

**Figure 5.**
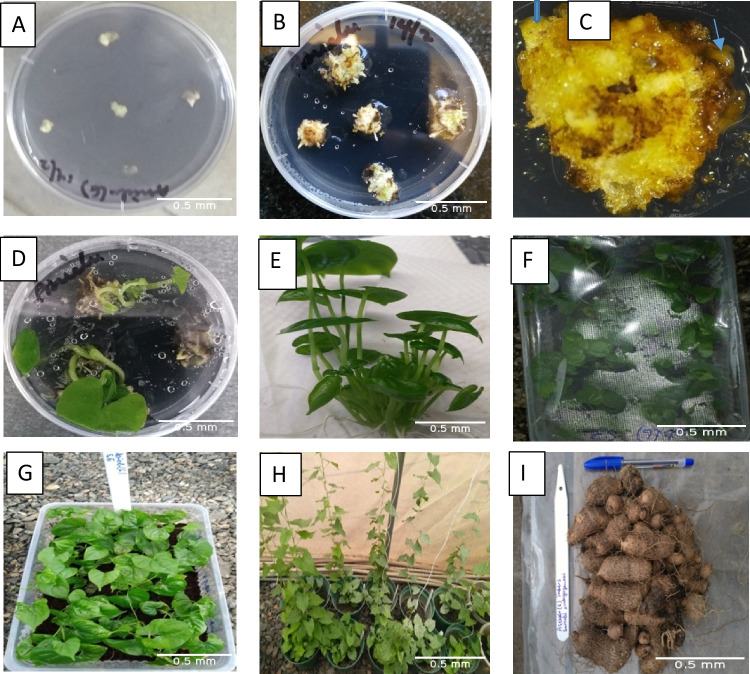
Stages of seed *Dioscorea rotundata* Poir. and *Dioscorea alata* L. tuber production *via* somatic embryogenesis in Asiedu. (*A*) Callus induction from the axillary bud (AB) explant at 2 wk of culturing (WOC). (*B*) Callus cultures at 4 WOC. (*C*) Somatic embryos at 6 WOC. (*D*) Regenerated plantlets at 10 WOC. (*E*) Regenerated plantlets at 17 WOC. (*F*) Hardening of plantlets at 17 WOC. (*G*) Hardened plantlets at 19 WOC. (*H*) Potted *in vitro* plantlets at 27 WOC. (*I*) Harvested tubers at 45 WOC. *Arrow*: Torpedo-shaped somatic embryo.

There was gradual increase in the number of nodes produced by all genotypes in both organogenesis and somatic embryogenesis from the 2nd to 8th WAP (Fig. 3). At 8 wk of potting, the NON produced by SE-raised plantlets in Kpamyo, Ekiti2a, and Asiedu were 8%, 26%, and 28% higher than the organogenesis-raised plantlets, respectively. However, in TDa2014 and Swaswa, the NON produced by the organogenesis-raised plantlets were 4% and 13% higher than the SE-raised plantlets, respectively. There was 100% survival of the somatic embryogenesis–produced plantlets after hardening in all genotypes while in the organogenesis-raised plantlets, all the plantlets introduced to hardening survived except for Asiedu and Kpamyo that had 90% and 80% survivals, respectively (Fig. 4*A*). The number of tubers by the organogenesis-raised plantlets after 7 mo of potting in Kpamyo, Ekiti2a, Asiedu, TDa2014, and Swaswa was 6%, 27%, 42%, 16%, and 8% higher than the SE-raised plantlets, respectively. The number of tubers by the organogenesis-raised plantlets after 7 mo of potting in Kpamyo, Ekiti2a, Asiedu, TDa2014, and Swaswa was 6%, 27%, 42%, 16%, and 8% higher than the SE-raised plantlets, respectively. In addition, the weight of tubers harvested from the organogenesis-raised plantlets after 7 mo of potting in Ekiti2a, Asiedu, TDa2014, and Swaswa was heavier than the SE-raised plantlets by 52%, 4%, 54%, and 11%, respectively. However, in Kpamyo, the weight of tuber produced by the SE-raised plantlets was 21% heavier than the organogenesis-raised plantlets (Fig. 4*B*). The sequence of activities (stages of regeneration) in the SE system started from the induction of embryogenic callus from axillary bud explant, followed by the production of somatic embryos and the maturation and production of plantlets from the somatic embryos (Fig. 5*A*–*C*). The plantlets obtained *via* SE and organogenesis were successfully acclimatized and potted. This led to the production of minitubers at 7 mo after potting from the plantlets obtained from both propagation pathways (Figs. 4 and 5).

## Discussion

Somatic embryogenesis is one of the fastest means of crop propagation because each somatic cell is capable of producing somatic embryos that are bipolar in nature (Junaid *et al.* 2013), permitting large numbers of reproductive units per explant. However, the success of SE is controlled by factors, such as genotypes, explant parts and age, and plant growth regulators (PGRs) (Fehér, 2008). The optimization of PGRs for the three basic stages of SE (initiation and proliferation of embryogenic callus, formation and maturation of somatic embryos, and plantlet regeneration) is very essential for the successful regeneration of plantlets from any somatic explant (Andrei and Peter 2014). In this study, at least 90% of the axillary bud explants initiated callus cultures in the three white yam genotypes within 2 to 3 wk of culture. This was two times higher than the two water yam genotypes, which had maximum of 50% callus induction rate. The successful induction of callus at 2 wk of culture supported the findings of Suarez *et al.* (2011) who worked with another improved *D. rotundata* genotype using the leaf explant. However, their callus induction frequency was low (< 30%). The low rate of callus proliferation in the water yam genotypes could be due to the genotype by culture environment interactions that cause dieback of cultures, a situation earlier reported in callus culture of water yam (Belarmino and Gonzales 2008).

In this study, the plantlet regeneration process proceeded from the induction and proliferation of embryogenic callus in an auxin-based medium to the formation and maturation of somatic embryos maintained in a PGR-free yam multiplication medium (YMM) and thereafter transferred to a cytokinin-based yam regeneration medium. These steps were successful in all the white and water yam genotypes evaluated with the landrace (Ekiti2a) having the highest number of plantlets regenerated. Organogenesis remains the most common means of propagating yam *in vitro* as SE has not been fully optimized for yam (Andrei and Peter 2014). The propagation ratios obtained through organogenesis in this study agree with the findings of Ondo *et al.* (2007) and Balogun *et al.* (2014); however, the organogenesis process is limited to the number of nodes in the mother plant whereas SE has been achieved using the leaves, stem, root, and axillary bud explants (Manoharan *et al.* 2016; Ossai *et al.* 2018). When these explants from one plant are factored in, an estimated 50 explants per plantlet of four nodes can be cultured simultaneously in an optimized protocol to give on average a propagation ratio of 1:300 in 8 wk or 1:800 in 16 wk of culture.

Since the regenerative pathways of organogenesis and somatic embryogenesis are difficult to differentiate morphogenically, and reports of both direct and indirect somatic embryogenesis occurring in same culture exist, the use of a histological study to identify the initiation of embryo-like structures becomes imperative (Gaj 2004). The histological observation of the cultured explants showed that at the callus proliferation phase, there was no definite tissue arrangement with dense cytoplasm. According to Ramos *et al.* (2014), the situation might be a result of active cell proliferation in an auxin medium, and it shows the origin of acquisition of embryogenic competence (Kamnoon and Preamudee 1999). However, at later stages (somatic embryo formation and plantlet regeneration) after the withdrawal of auxin in the culture medium, the tissue arrangement pattern revealed the vascular bundles (xylem and phloem). The numerous centers of meristematic arrangements, with cells in the center smaller than the outer layers, were more vacuolated which corresponds to the initiation of somatic embryos, embryo maturation, and plantlet regeneration stages. According to Kamnoon and Preamudee (1999), this center differentiates into embryoids that form plantlets. This finding is further validated by the presence of torpedo-shaped somatic embryos and plantlets regenerated as early as six (6) wk of culture during the confirmation of embryo formation. However, at 6 WOC, different stages of embryo formation were developed in a callus mass (Fig. 4*C*). This shows that the stages of embryo formation are variable within and between cultures.

In yam, *in vitro* propagation is mostly through organogenesis (Balogun and Gueye 2013). Nonetheless, somatic embryogenesis could be more proficient because all somatic embryos can potentially regenerate into a whole plant (Mousavizadeh 2009). Unlike organogenesis that produces a unipolar seed, somatic embryogenesis produces a bipolar seed and vascular tissues without connection with the parent tissue (von Arnorld *et. al*. 2002), allowing a large number of reproductive units having root and shoot meristems per culture. The somatic embryogenesis process can be achieved in direct or indirect processes. In the direct process, embryos are formed from pre-embryonic cells with the embryos attached to the initial explant, thereby creating identical clones, while in the indirect process, they are formed from callus, an unorganized tissue that is formed from the initial explant tissue (Nakamura *et al*. 1991). Identifying the period of acquisition of vascular tissues from an undifferentiated mass of cells that is not attached to the initial explant after withdrawal from an auxin-rich medium (such as 2,4-D- and NAA-supplemented medium) is important in elucidating the regeneration pathway as the attainment of vascular tissue arrangement that corresponds to the embryo development and coordination (Lucas *et al.* 2013).

Above 80% of plantlet survival after acclimatization was achieved in this study for both organogenesis- and somatic embryogenesis–raised plantlets. The hardened plantlets also established well in pots irrespective of the propagation mode, and both vines and seed tubers were produced of which Asiedu showed superior growth and yield (tuber production) performances relative to other genotypes. Both micropropagation pathways (SE and organogenesis) remain incomplete until the *in vitro* plantlets have been successfully hardened because there could be 100% loss of *in vitro* produced plantlets in post-flask if the acclimatization process is not adequately optimized (Deb and Imchen 2010). The survival of the *in vitro* produced plantlets within 2 wk of acclimatization suggests that the roots formed in the culture medium may have enhanced the continued growth and development of the plants in hardening (Alfred and Uchenna 2013). The variety ‘Asiedu’, which is the new name registered for Breeders’ line ‘TDr 89/02665’, has a high survival rate through rooted single-node vine cuttings at 90 d after cutting (Maroya *et al.* 2014). Agbaje *et al.* (2003) also reported a higher tuber yield from Asiedu against other improved yam genotypes they tested. The successful production of tubers (post-flask) from plantlets propagated through organogenesis and SE shows that the two systems can be used effectively in the multiplication of yam *in vitro*. However, with reports of somaclonal variations discovered in potatoes propagated through somatic embryogenesis, it is important to further screen the regenerants obtained through somatic embryogenesis for their true-to-type with the mother plants for full-scale production of clean breeder seed yam for commercial purposes.

In conclusion, the findings of this study have validated the genotype-dependent responses of yam to somatic embryogenesis, which poses a challenge in the adoption of the system in yam multiplication against the more optimized organogenesis protocol. In addition, the best propagation rates in the genotypes were pathway-dependent as some genotypes had higher nodal production *via* SE while others *via* organogenesis. The axillary bud explant was effectively used in this study to propagate yam through somatic embryogenesis, and it could be a viable alternative to the propagation of yam relative to organogenesis as a result of the higher number of culture explants as against the organogenesis process that is limited to the meristematic parts. The regenerants through both organogenesis and somatic embryogenesis were successfully acclimatized, and yam tubers were produced from them.

## Data Availability

All data used during the study are available from the author Ossai Chukwunalu Okolie by request (c.ossai@cgiar.org).
